# The regulation of mitochondrial ferritin on mitochondrial redox balance is essential to cell fate decision

**DOI:** 10.1016/j.redox.2026.104124

**Published:** 2026-03-11

**Authors:** Yuanyuan Liu, Yan-Zhong Chang

**Affiliations:** Laboratory of Molecular Iron Metabolism, Key Laboratory of Molecular and Cellular Biology of Ministry of Education, Hebei Key Laboratory of Animal Physiology, Biochemistry and Molecular Biology, Hebei Collaborative Innovation Center for Eco-Environment, Hebei Research Center of the Basic Discipline of Cell Biology, College of Life Sciences, Hebei Normal University, Shijiazhuang, Hebei Province, 050024, China

**Keywords:** Mitochondrial ferritin, Redox, Iron, Cell death, Neurodegenerative disease

## Abstract

Mitochondrial ferritin (FtMt), first identified by Levi et al., is an iron-storage protein with high homology with cytoplasmic ferritin. It is mainly expressed in metabolically active tissues and exhibits distinct physiological and biochemical properties compared cytoplasmic ferritin. Over the past few decades, significant attention has been drawn to the unique structural and functional characteristics of FtMt that differentiates it from conventional ferritin. Mitochondrial ferritin specifically located on the mitochondrial exhibits unique advantages in mitochondrial redox balance through isolating iron within mitochondria, reducing oxidative stress and maintaining mitochondrial homeostasis. Moreover, it modulates the labile iron pool within mitochondria, facilitating the biosynthesis of iron-sulfur clusters and supporting cellular respiration. This review comprehensively discusses the pivotal function of FtMt in regulating mitochondrial redox homeostasis and its impact on cell fate decisions, specifically, its influence on apoptosis, ferroptosis through alterations in mitochondrial integrity. We also summarize recent advances in understanding the association between FtMt dysregulation and various diseases, emphasizing its implications in neurodegenerative diseases, cardiovascular disorders and cerebrovascular pathologies. By critically evaluating emerging evidence, this article aims to provide translational insights into targeting FtMt and mitochondrial redox homeostasis as therapeutic strategies for mitigating these clinically significant diseases.

## Highlights

• Mitochondrial ferritin (FtMt) can isolate free iron in mitochondria and participate in the regulation of mitochondrial redox balance.
• FtMt participates in cellular energy generation and mitophagy processes.
• FtMt participates in cell fate determination by regulating cellular redox balance.
• FtMt plays an important protective role in various neurodegenerative, tumor, and aging-related diseases.
• FtMt provides new therapeutic strategies for maintaining redox balance and disease intervention.

## 1 Introduction

Mitochondria make a significant contribution to the entire life process of cells. They generate ATP via oxidative phosphorylation, supplying energy for various cellular activities, which positions them at the core of energy metabolism and as a central hub of metabolic activity. Based on the unique physiological function of mitochondria in cells, electrons in the respiratory chain combine with oxygen molecules to generate superoxide, and the “missed” electrons in the respiratory chain can combine with oxygen molecules to form superoxide [1,2]. This implies that higher energy production is accompanied by an increased generation of oxidants. Thus, maintaining mitochondrial redox balance is crucial while sustaining efficient ATP synthesis.

Multiple diseases are associated with mitochondrial redox imbalance, including Alzheimer's disease, Parkinson's disease, ischemic stroke, heart disease and cancer etc [[3], [4], [5], [6]]. This dysregulation typically involves excessive accumulation of oxidants within mitochondria, resulting in mitochondrial dysfunction and ultimately triggering cell death. Alterations in mitochondrial function directly influence cellular fate, determining whether cells undergo apoptosis, necrosis, ferroptosis, and autophagy. Consequently, growing research attention is being directed toward understanding and regulating mitochondrial redox homeostasis.

Iron has consistently served as a double-edged sword in mitochondriabiology. As a central regulatory element, iron directly or indirectly engages in multiple critical physiological processes within mitochondria-including energy generation, substance metabolism, and the maintenance of redox homeostasis. It also exerts a decisive influence over cell fate decisions, such as ferroptosis and autophagy. Mitochondrial iron is incorporated into iron-sulfur (Fe–S) clusters and heme groups, which act as essential cofactors in the electron transport chain complexes. Moreover, they are integral components of key enzymes in the tricarboxylic acid (TCA) cycle, including succinate dehydrogenase and aconitase. The conversion of iron ion valence states directly participates in electron transfer in cytochromes. Therefore, the maintenance of normal mitochondrial function relies on the involvement of iron. However, the valence transitions that enable electron transfer also promote the generation of reactive oxygen species (ROS). Furthermore, within the highly metabolically active milieu of mitochondria, excess ferrous ions can catalyze the Fenton reaction, producing various oxygen free radicals. This exacerbates oxidative stress, disrupts redox balance, and compromises mitochondrial integrity and function. Thus, stringent regulation of iron homeostasis within mitochondria is imperative.

The discovery of mitochondrial ferritin (FtMt) has provided a novel strategy to address this issue. Unlike the structurally and functionally similar cytoplasmic ferritin heavy chain (FtH) [7,8], FtMt is localized within mitochondria. On one hand, it maintains mitochondrial iron utilization and regulates the levels of bioavailable iron in mitochondria, thereby preventing redox imbalance and preserving normal physiological functions. On the other hand, it facilitates the preferential translocation of cytoplasmic iron into mitochondria, sequesters excess free iron that may cause damage, and modulates the cellular labile iron pool (LIP), thus helping to maintain cellular redox homeostasis. Therefore, FtMt not only plays an essential role in preserving free iron levels in cells and mitochondria and ensuring their normal physiological functions, but also is critical for protecting mitochondria and cells from iron-induced oxidative damage and determining cell fate [[9], [10], [11]]. In vivo, FtMt is specifically enriched in tissues with high metabolic activity, such as the brain, heart, and testes [12]. These characteristics indicate that the main function of FtMt is not merely to store iron, but to maintain the redox balance within mitochondria and cells, and protect high-oxygen-consuming tissues and cells from iron-dependent oxidative damage. Previous studies have demonstrated that FtMt plays a protective role in various iron-related disorders, including Alzheimer's disease (AD), Parkinson's disease (PD), and heart damage [[13], [14], [15], [16], [17]]. In this review, we systematically discuss the role of FtMt in preserving and modulating redox balance in cells fate determination and the pathogenesis of diverse diseases.

## 2 FtMt is involved in mitochondrial homeostasis and physiology

### 2.1 The dual role of iron in mitochondria

Mitochondria function as the cellular powerhouse and are the primary sites of ATP production through oxidative phosphorylation. Iron, as an essential key element, participates in the entire process of cellular life activities and is also a core substrate for mitochondrial energy metabolism [18]. It participates in the formation of mitochondrial electron transport chain (ETC) complexes and key metal cofactors for ATP synthase [19]. Notably, a large number of Fe–S clusters and heme groups within the respiratory chain are iron-centered [19]. Iron completes electron transfer through the transition of redox states, which is a necessary condition for mitochondria to synthesize ATP through oxidative phosphorylation. The core value of iron lies in its chemical properties: iron atoms have variable redox states and can participate in redox reactions as electron donors or acceptors. Simultaneously, iron binds to various functional proteins as metal cofactors, helping to maintain protein conformation and activity [20].

Due to the unique iron requirements of mitochondria and the process of energy production, mitochondria serve as the primary source of intracellular ROS [21]. Multiple cellular reactions and signaling pathways significantly contribute to the formation of ROS, including the mitochondrial respiratory chain, the TCA cycle, the Fenton reaction and lipid oxidation [22,23]. In particular, complexes I and III of the ETC are prone to electron leakage, which leads to the formation of superoxide anions upon interacion with molecular oxygen [[24], [25], [26]]. Notably, electron transfer within the ETC depends not only on protein complexes containing Fe–S clusters or heme but also on the redox cycling of iron between different valence states. The inherent chemical properties of iron allow it to exist in multiple oxidation states. Among these, the interconversion between Fe^2+^ and Fe^3+^ is the most prevalent in biological systems and plays a key role in essential physiological processes such as mitochondrial electron transfer [27,28]. However, this same redox cycling also creates a platform for the generation of oxygen radicals [29]. When Fe^2+^ is oxidized to Fe^3+^, a released electron can support mitochondrial electron transfer and sustain normal physiological metabolism. On the contrary, if this reaction occurs in the presence of H_2_O_2_, hydroxyl radicals (•OH) are produced via the Fenton reaction [30]. In the metabolically active environment of mitochondria, electrons released during the Fe^2+^ to Fe^3+^ transition can also combine with oxygen to form peroxide anions, which may further give rise to •OH through the Haber Weiss reaction, thereby accelerating ROS production and perpetuating a redox cycling [31,32].

Therefore, the interconversion of iron valence states is crucial for maintaining redox balance in mitochondria. Intracellular Fe^2+^ must be tightly regulated and spatially sequestered to prevent oxidation damage to cell resulting from its valence transitions. Consequently, strictly control the level of Fe^2+^ in mitochondria at the source represents a fundamental strategy for modulating mitochondrial peroxide production.

### 2.2 FtMt is a potential intracellular antioxidant pathway

**Fig. 1:**
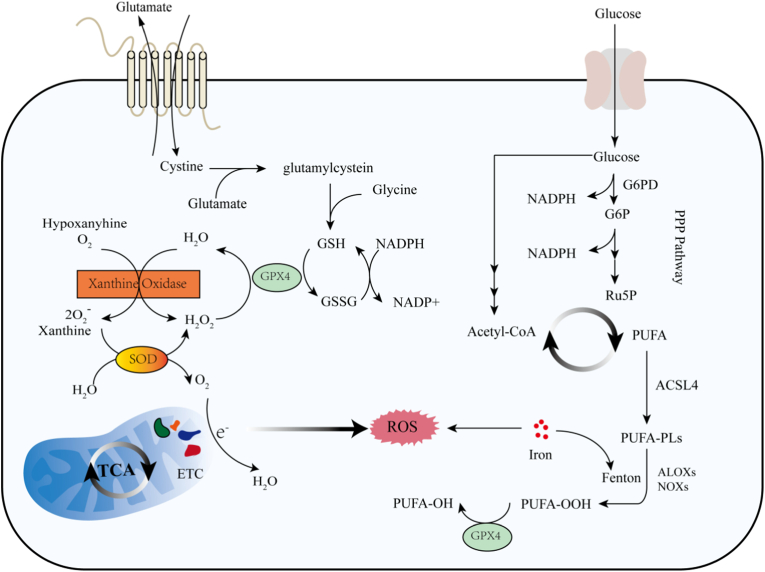
ROS production and antioxidant balance in cells. Cellular metabolites and mitochondrial respiration contribute to intracellular ROS production. The acetyl-coA produced by the metabolism of PUFA and glucose participates in the TCA cycle, and the generated NADH and FADH_2_ participate in electron transport in the mitochondrial respiratory chain, some of which leak and react with O_2_ to form superoxide radical O_2_^−^. PUFA, under the action of related enzymes, generates PL-OOH through the iron and Fenton reaction, which promotes ROS production. Xanthine oxidase catalyzes the oxidation of hypoxanthine to xanthine, which simultaneously generates superoxide anion and H_2_O_2_. SOD can reduce O_2_^−^ to H_2_O_2_ and O2, and further, GPX4 can reduce H_2_O_2_ to H_2_O, and NADPH generated by PPP provides reducing power for GSH. System Xc^−^ transports cysteine into cells, where it is synthesized into glutathione along with glycine and glutamate. This process requires the participation of the reducing agent NADPH. Together, these constitute important antioxidant systems within the cell.

The intracellular antioxidant system is mainly composed of superoxide dismutase (SOD), catalase, and glutathione peroxidase (GPX) [33,34] (Fig. 1). Multiple metals participate in the composition of SOD as cofactors, among which Manganese Superoxide Dismutase (MnSOD) is mainly responsible for the clearance of superoxide in mitochondria [35], which can catalyze the superoxide anions generated in the respiratory chain into H_2_O_2_ [[36], [37], [38], [39]]. Another important antioxidant molecule in cells is the GPX family. The GPX family consists of multiple members, among which GPX1 and GPX4 are involved in the clearance of mitochondrial ROS [40]. GPX1 reduces H_2_O_2_ to using reduced glutathione (GSH) [41], while GPX4 can specifically remove membrane-bound lipid peroxides [42,43]. The non-enzymatic antioxidant GSH also plays an important role in ROS scavenging [44]. When the damage is merely local lipid peroxidation, such as the generation of lipid free radicals caused by a small amount of iron ion leakage, GPX4 can efficiently eliminate lipid hydroperoxides, protecting the integrity of mitochondrial and cell membranes [43]. However, when the damage arises from the disruption of mitochondrial metabolic pathways, such as cysteine deprivation, the underlying cause is metabolic imbalance rather than merely the accumulation of lipid peroxides. In this case, GPX4 cannot effectively suppress ferroptosis. Studies have shown that cysteine deprivation leads to abnormal mitochondrial membrane potential and substantial accumulation of lipid peroxides [45,46]. The oxidative damage in this process represents systemic injury driven by mitochondrial metabolic dysfunction. Thus, in ferroptosis triggered by abnormal mitochondrial metabolic pathways, the role of GPX4 is limited.

Although the GPX system plays an irreplaceable role in scavenging ROS, blocking lipid peroxidation chain reactions and inhibiting ferroptosis. Its core function lies in reducing peroxides such as H_2_O_2_ and lipid peroxides, it does not directly neutralize free radicals. •OH are the main source of damage in iron-dependent oxidative stress [47]. This means that GPX system acts at a downstream stage of the lipid peroxidation chain reaction, rather than the initiation stage. Furthermore, the function of GPX system is highly dependent on GSH, selenium and NADPH, which are essential for maintaining its active center [42,48]. Therefore, the function of the GPX system is not omnipotent. It has limitations such as substrate specificity, cofactor dependence, and an oxidative stress threshold. These constraints determine that it cannot counteract all forms of oxidative damage, especially under conditions of severe oxidative stress environment caused by mitochondrial iron overload, where its protective effect will quickly saturate or even fail.

In this context, we focus on mitochondrial ferritin, a protein located on the mitochondrial membrane that can chelate iron in mitochondria and has iron storage function [7]. Previous studies have shown that mitochondrial ferritin can reduce ROS generation and oxidative damage during H_2_O_2_ or hypoxia by isolating iron in mitochondria [10,[49], [50], [51]]. FtMt directly blocks the occurrence of Fenton reaction by chelating mitochondrial LIP (mtLIP), reducing the generation of •OH from the source and decreasing their accumulation rate, although it cannot directly eliminate the generated lipid peroxides. The GPX system can remove residual peroxides, achieving thorough removal of oxidative damage.

In summary, the FtMt and GPX systems have irreplaceable complementarity in oxidative stress defense, and they play their unique roles in different oxidative damages. In iron-dependent oxidative stress, FtMt plays a central role, while GPX4 can eliminate the lipid peroxides that has already been produced and block lipid peroxidation from the end. Both are indispensable in maintaining cellular redox homeostasis.

### 2.3 FtMt regulates the availability of iron within cells

The cage-like structure and ferroxidase activity of FtMt enable its unique physiological function: oxidizing Fe^2+^ to Fe^3+^ and storing it within its cavity. FtMt can regulate the availability of cellular iron, allowing the iron in the cells to preferentially enter the mitochondria. In an early study, Corsi et al. expressed wild-type FtMt in HeLa cells and labeled it with ^55^Fe [52]. Both mitochondrial-localized FtMt and mitochondrial-targeted H-Ferritin incorporated ^55^Fe, whereas ferroxidase-defective FtMt mutants lost iron uptake capacity. Overexpression of FtMt in mitochondria reduced cytoplasmic ferritin levels and increased expression of the iron uptake protein Transferrin Receptor (TfR) [52]. Interestingly, treatment with the iron chelating agent Deferoxamine (DFO) diminished FtMt's iron-sequestering ability, suggesting that FtMt modulates intracellular iron availability. Nie et al. Further investigated the role of FtMt in cellular iron metabolism [9]. Overexpression of FtMt in human H1299 cells decreased cytoplasmic ferritin and LIP, resulting in cellular “iron starvation”. This was accompanied by increased binding and uptake efficiency of transferrin-bound iron (Tf-Fe). Using ^59^Fe labeling, they demonstrated that iron was preferentially distributed to mitochondria in FtMt-overexpression cells, consistent with the findings of Corsi et al. These studies collectively confirm that FtMt participates in mitochondrial iron storage and modulates cellular iron availability. However, it remains unclear how FtMt mobilizes intracellular iron to enter mitochondria preferentially. Additionally, FtMt overexpression reduces the proportion of heme iron in cells [9].

Although FtMt is known to possess ferroxidase activity, the detailed process of Fe^2+^ entering the FtMt interior and aggregating to form nuclei after oxidation remains poorly understood due to the lack of high-resolution FtMt protein structure data. Bradley et al. provided new insights into this process in a recent study published in JACS [53]. They used a new method to obtain iron-containing FtMt protein crystals and analyzed and characterized the internal nucleation sites and iron-oxygen clusters using X-rays. To observe the process of Fe^2+^ oxidation and nucleation within FtMt, the authors first prepared Fe^2+^/Mg^2+^-containing FtMt crystals under anaerobic conditions and then placed them in an aerobic environment. X-ray diffraction determined that the glutamic acid residues E61 and E64 on the inner surface of FtMt are important sites for the oxidation of Fe^2+^ and nucleation within FtMt [53]. Among them, the glutamic acid at the E61 site plays a key role in iron nucleation within FtMt. After replacing this site with alanine, it was found that it did not affect the oxidation of Fe^2+^ at the active center, but the rate of Fe^3+^ release from the active center was significantly reduced, indicating that the E61 residue is crucial for the process of Fe^3+^ leaving the active center and nucleation.

### 2.4 Mitochondrial ferritin maintains mitochondrial redox balance and regulates free radical levels by modulating the labile iron pool within mitochondria

Iron is indispensable for mitochondrial function, where it supports various physiological processes and the synthesis of biomolecules. However, iron can also contribute to mitochondrial ROS production and cellular damage through multiple mechanisms. Owing to its capacity to redistribute and compartmentalize iron fluxes between the cytoplasm and mitochondria, FtMt plays an important role in mitigating iron overload-related pathologies and oxidative damage.

Previous studies have also shown that high expression of FtMt rescues H_2_O_2_ and Erastin induced cell death [49,50]. At the same time, treatment with a certain concentration of oxidant H_2_O_2_ also upregulates FtMt expression, suggesting a close relationship between FtMt and cellular oxidative balance [50]. However, the regulatory mechanism of oxidants on FtMt is still unclear. Many studies have confirmed that FtMt can regulate the cellular free iron pool, allowing cytoplasmic iron to preferentially enter mitochondria, reducing free iron in cells and mitochondria, and thereby reducing oxidative stress damage [9,54], which has been shown to play a protective role in apoptosis and ferroptosis [51,55]. The absence of frataxin in yeast and HeLa cells leads to Friedreich ataxia (FRDA), which is characterized by mitochondrial iron accumulation and oxidative damage [10,56,57]. Overexpression of FtMt has been shown to rescue respiratory defects resulting from frataxin deficiency, prevent mitochondrial iron overload, partially protected the activity of mitochondrial Fe–S enzymes, and enhanced cell resistance to H_2_O_2_ [56,58]. Similarly, in HeLa cells, the expression of FtMt improved cell survival under oxidative stress, antimycin A, and glucose deprivation, while attenuating oxidative damage. By regulating the mitochondrial LIP, FtMt helps maintain the activity of coenzyme, prevents ATP depletion. Comparable protective effects were observed in fibroblasts derived from FRDA patients upon the expression of FtMt [59]. Numerous studies across different disease models and injury contexts indicate that overexpression of FtMt reduces oxidative damage by regulating the availability of iron within cellular and mitochondrial compartments [17,51,60,61]. In summary, FtMt regulates the availability of iron, centralizes the storage of mitochondrial iron, and prevents oxidative damage caused by excessive free iron through processes such as Fenton reaction.

### 2.5 FtMt participates in the synthesis of Fe–S clusters and mitochondrial respiration

**Fig. 2:**
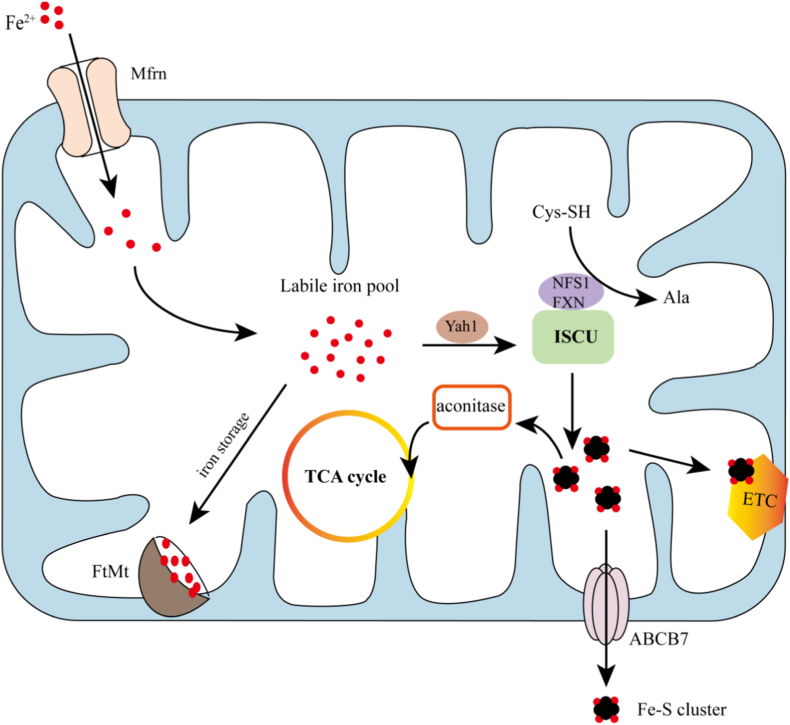
Iron metabolism process in mitochondria. Mitochondria, as independent iron metabolism units, have their own specific iron metabolism patterns. Fe^2+^ in the cytoplasm enters the mitochondrial matrix through the mitochondrial membrane proteins Mfrn (including Mfrn1 and Mfrn2), where Fe^2+^ aggregates to form mtLIP. The remaining Fe^2+^ is stored in mitochondrial ferritin to prevent excessive Fe^2+^ induced oxidative damage. Fe^2+^ entering mitochondria is mainly used for the synthesis of heme and Fe–S clusters. Among them, the mitochondrial scaffold protein ISCU provides a site for the synthesis of Fe–S clusters. After the synthesis of Fe–S clusters, they will participate in the assembly of corresponding target proteins, and some will be transferred to the cytoplasm through mitochondrial membrane proteins ABCB7. Fe–S clusters participate in the formation of various complexes on the electron transfer chain, as well as enzymes in the TCA cycle, playing a crucial role in electron transfer and cellular metabolism. Therefore, FtMt indirectly affects iron sulfur clusters and cellular metabolism by regulating mitochondrial LIP levels.

Mitochondria function are not only as the energy centers of cells, participating in the synthesis and metabolism of various biomolecules such as amino acid fatty acid metabolism, but also play a vital role in synthesis of heme and Fe–S clusters [62,63]. They serve as essential sites for the production of both heme and Fe–S clusters [64,65]. Heme, an iron-containing prosthetic group, is a critical component of hemoglobin and various cytochromes. It plays indispensable roles in oxygen transport, storage, and electron transfer processes [[66], [67], [68]]. Heme biosynthesis primarily occurs in erythroid cell and involves a multi-step pathway. The process initiates in mitochondria with the formation of δ-aminolevulinic acid which is then exported to the cytoplasm and converted into protoporphyrin IV through a series of enzymatic reactions [69]. The final step takes place back in mitochondria, where ferrochelatase catalyzes the incorporation of Fe^2+^ into protoporphyrin IX to form heme [70,71]. Fe–S cluster is an important iron containing compound that serves as a cofactor in the composition of many enzymes and participates in physiological processes such as electron transfer and enzymatic reactions [[72], [73], [74]]. Mitochondria are the key sites for the synthesis of the Fe–S cluster within eukaryotic cells, and they are the starting point and assembly site for Fe–S synthesis [75] (Fig. 2). The biosynthesis process of the Fe–S clusters is highly coordinated and involves multiple proteins and steps. Briefly, the cysteine desulfurase NFS1 provides sulfur atom derived from cysteine [76], while the iron chaperone protein Yfh1 supplies iron atoms. With the support of additional auxiliary proteins [77], the Fe-s clusters are assembled [78,79]. The scaffold protein ISCU located in the mitochondrial matrix provides support for the synthesis of Fe–S clusters [80], and the core components of Fe–S are mainly assembled and synthesized on ISCU [81]. Variations in iron and sulfur stoichiometry give rise to different types of Fe–S clusters, such as 2Fe–2S, 4Fe–4S, etc., and they participate in different physiological processes [82].

Iron deficiency induces the activation of the iron response element/iron response protein (IRE/IRP) system, enhancing the binding activity between IRE and IRP, but the total IRP activity remains unchanged [9]. Meanwhile, the activities of aconitases with the same structure as IRP were inhibited, including those in the cytoplasm and mitochondria, as they were affected due to the presence of the 4Fe–4S cluster. This result is contrary to the research of Campanella and Sutak et al. Campanella et al. found that under the stimulation of H_2_O_2_ and antimycin A treatment, the expression of FtMt can reduce ROS generation, increasing succinate dehydrogenase and mitochondrial uricase activity [10]. Similarly, the expression of FtMt partially restored mitochondrial uricase activity in fibroblasts of FRDA patients. Sutak et al. also reached similar conclusions in their study on yeast, stating that the expression of FtMt in frataxin-deficient yeast increases Fe–S enzyme activity and promotes heme synthesis [58]. This result suggests that FtMt plays a protective role under certain conditional stimuli, such as respiratory defects or oxidative stress.

## 3 Characteristics of mitochondrial ferritin

### 3.1 Molecular structure and distribution of mitochondrial ferritin

In 2001, Levi et al. discovered and reported FtMt in humans. It is a single copy gene located on chromosome 5q23.1 and can encode a precursor protein containing 242 amino acids, approximately 30 kDa [7]. Its N-terminus contains a leading peptide with a spacer arginine sequence, which can specifically target mitochondria, where FtMt is cleaved and processed into a mature protein of 22 kDa size [7]. FtMt sequences are similar in different species including human, mouse and *Drosophlia* [83]. It is worth noting that the FtMt gene lacks introns, whereas FtH contains multiple introns. Meanwhile, FtMt exhibits high homology with the sequence of FtH, suggesting that FtMt may have originated from the reverse transcription of FtH mRNA. Additionally, FtMt does not contain an IRE sequence, while the 5’ end of FtH mRNA contains an IRE precisely within its untranslated region, which is consistent with a reverse transcription origin. Taken together, FtMt is likely derived from reverse transcription events of FtH mRNA.

To date, FtMt expression has been identified only in mammals, *Drosophila*, and some plants [83,84] From an evolutionary perspective, FtMt is not a widely conserved gene and therefore cannot be regarded as an ancient or essential component of the iron metabolism pathway. Indeed, some organisms that lack the FtMt-coding sequence are still capable of carrying out mitochondria iron metabolism and energy generation through alternative mechanisms. However, its restricted phylogenetic distribution does not diminish its functional relevance. On the contrary, this pattern implies that FtMt likely emerged as a specialized regulatory adaptation in lineages experiencing severe mitochondrial oxidative stress and iron burden, rather than being necessary for core mitochondrial functions. FtMt represents an adaptive protein that enhances cellular resilience under stress conditions, emphasizing its significant role in the evolutionary adaptation of specific organisms to metabolic and oxidative challenges.

The expression of FtMt in the body also exhibits tissue specificity. In human, FtMt is mainly highly expressed in the testes, followed by a small amount of expression in tissues such as the heart, brain and kidney [7,11,12,85]. It has been found that FtMt is significantly upregulated in eyrythroid cells of sideroblastic anaemia patients [11,86]. In mice, it was found that FtMt was mainly expressed in testicular interstitial cells and mature sperm within the seminiferous tubules [12]. The predominant expression of FtMt in the testes is primarily driven by the unique physiological demands of this organ. This represents a precise adaptation strategy aimed at reducing cellular and mitochondrial oxidative damage while supporting iron requirements and energy metabolism, providing guarantees for spermatogenesis. The testes sustain continuous spermatogenesis, a process involving rapid cell proliferation, extensive DNA replication, and intense energy metabolism. Consequently, cells require more iron to support DNA replication and enzymatic reactions as a cofactor while mitochondria must enhance respiratory activity and energy output. This heightened metabolic state, however, also raises the risk of ROS generation via the Fenton reaction. To ensure the demand for high-speed rail and energy metabolism, as well as lower ROS damage, cells need a unique mechanism to reduce the risk of injury. FtMt serves this role by sequestering iron within mitochondria, thereby limiting lipid ROS formation through Fenton reaction, and ensures normal energy metabolism. Unlike other tissues, spermatogenesis in the testes is a unique and precise process, and any DNA or cellular damage can lead to genetic problems in offspring, requiring stricter protective mechanisms.

The tissue-specific expression pattern of FtMt further indicates that not all cell types depend on this protein. In the seminal study by Bartnikas et al., FtMt-knockout mice exhibited no alterations in systemic iron parameters (serum and liver iron levels) or erythrocyte indices, and no obvious reproductive phenotype was observed in males [87]. In contrast, Maccarinelli et al. reported that FtMt deficiency in male mice showed reduced testicular weight, lower sperm counts, and diminished reproductive success in mating trials, with no evident abnormalities in sperm morphology [88]. These findings suggest that FtMt may influence sperm development and maturation rather than sperm structure. It should be noted, however, that the relatively small sample size in the morphological analysis necessitates further experimental validation to clarify the role of FtMt in testicular physiology and resolve existing discrepancies in the literature.

FtMt is expressed in brain, while not all cells and brain regions express it. Synder et al. examined the distribution of FtMt in the brain of mice. They found FtMt is not primarily expressed in glial cells but mainly in cortical neurons, cerebellar Purkinje cells, and intraocular retina [12,89]. FtMt is expressed in multiple regions of the brain, such as the cortex, hippocampus, striatum, and cerebellum, but the most expressed FtMt is still concentrated in ependymal cells with a larger number of mitochondria [85,89].

Yang et al. prepared anti-human and anti-monkey antibodies that specifically recognize FtMt without cross reactivity with FtH to analyze the distribution of FtMt in the brainstem of macaques using immunohistochemistry and immunoblotting methods [90]. The results showed that FtMt was expressed in the extrapyramidal system, sensory trigeminal nucleus, some motor nuclei including the fuzzy nucleus, vagus dorsal motor nucleus, and sublingual nucleus, as well as some dorsal column nuclei such as the slender nucleus and wedge-shaped nucleus [89,90]. However, FtMt is not expressed in the liver, the main iron containing organ, but mainly in high metabolic tissue organs, indicating that FtMt may not be primarily responsible for intracellular iron storage and metabolism, but seems to be related to mitochondrial high energy demand.

### 3.2 Regulation of mitochondrial ferritin gene expression

The expression of FtMt is mainly regulated at the transcriptional level and post-transcriptional modification. Furthermore, some epigenetic modifications are also involved in the expression of FtMt. Although several studies have identified a limited number of transcription factors involved in the regulation of FtMt, the overall regulatory mechanism of FtMt remains incompletely understood.

The similarity of the FtMt promoter sequence between humans and macaques is 93%, while the similarity between mice is 70% [91]. Chromatin immunoprecipitation (ChIP) analysis of the FtMt promoter in K256 cells revealed transcription factors CREB, SP1, and YY1 bind to the positive regulatory region, while FoxA1, Gata2, and C/EBP bind to the negative regulatory region [91,92], which was further validated in tumor cells [93]. NF-kB, activated by different cytokines, can also significantly upregulate the expression of FtMt mRNA, and this upregulation shows certain temporal and dose-dependent characteristics in ARPE-19 or IMR-32 cells [94,95].

Hypoxia represents a critical regulator of FtMt. Wu et al. demonstrated in both in vivo and in vitro experiments that hypoxia significantly upregulated the expression of FtMt and hypoxia-inducible factor-1α (HIF-1α) [96]. By comparing the FtMt promoter sequences of humans, macaques and chimpanzees, the authors identified six hypoxia response elements (HREs), which are the binding sites for HIF-1α and the FtMt promoter [96].

However, in human macrophages, hypoxia induced FtMt expression relies on HIF-2α and requires serum derived thrombin for proteolytic maturation of FtMt precursors [97,98]. Hypoxia can downregulate the production of Nuclear Receptor Coactivator 4 (NCOA4) mRNA, while NCOA4 has been shown to directly inhibit FtMt expression [97]. Xu et al. explored the mechanism by which high glucose induces the upregulation of FtMt in cardiomyocytes (CM) and cardiac fibroblasts (CF) in their study on ferroptosis. High glucose treatment induces significant ferroptosis in CM, and at the same time, FtMt is significantly upregulated, suggesting that FtMt may be involved in ferroptosis induced by high glucose. ChIP identified FoxA1 as a key transcription factor mediating this response. High glucose upregulates the expression of FoxA1 and promotes its binding to the FtMt promoter region from −729 to −239, thereby activating transcription [99]. Collectively, these findings indicate that FtMt transcription diverse stimuli and cell type-specific factors, though potential crosstalk among these regulatory pathways under varying conditions remains to be elucidated.

Epigenetic mechanisms also contribute to FtMt regulation. UCSC analysis and prediction of the FtMt promoter revealed the presence of CpG islands, which are typically prone to methylation modifications and affect gene transcription activity [100]. Consistent with this, luciferase reporter assays in K256 and HeLa cells showed that both methylation inhibitors and histone deacetylation inhibitors upregulate gene expression [91]. Recently, Zhu et al. conducted a study on a mouse model of perinatal hypoxia and identified cold-induced RNA binding protein (CIRBP) as a key regulatory factor for FtMt [101]. Specifically, CIRBP binds to the 3′UTR of FtMt, delaying its mRNA degradation and thereby promoting the accumulation of FtMt [101]. This finding provides a novel post-transcriptional regulatory mechanism for FtMt and expands our understanding of its role in cellular iron homeostasis.

## 4 Comparative analysis of FtMt and FtH: structural, functional, and regulatory divergence

**Fig. 3:**
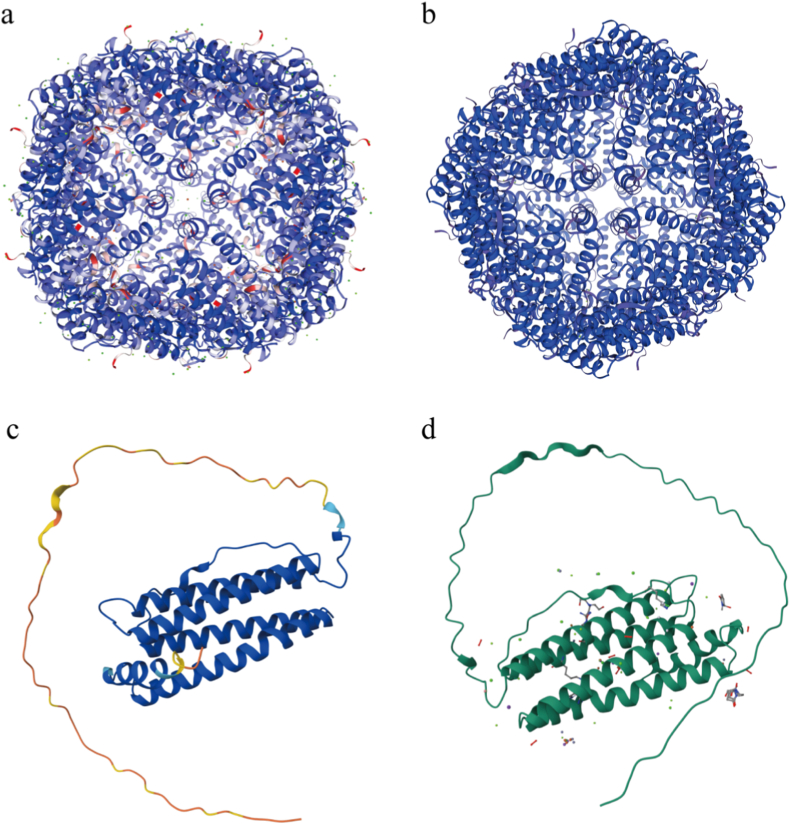
Mitochondrial ferritin structure in human and mouse. Mitochondrial ferritin 3-dimensional structure in humans (a) and mice (b). The 24 subunits make up the mature mitochondrial ferritin polymer, which appears as a spherical mechanism and can be used to store iron atoms inside. The molecular subunit structure of mitochondrial ferritin in humans (c) and mice (d), which has a four α-helix structure composed of two antiparallel α-helices. Its N-terminus has a long peptide chain that can target mitochondria, which will then be cleaved off. The structures were obtained from SWISS-MODEL and UniProt.

The molecular function and size of FtMt in human is very similar to the FtH [11,83]. It has 75% homology with FtH, but only 65% homology with FtL [8,11]. Both of them have similar structures, forming a hollow spherical shell for storing iron atoms (Fig. 3). The FtMt is assembled into a homologous polymer composed of 24 identical subunits, while the cytosolic ferritin is a heterologous polymer of 24 subunits, each consisting of an H chain and an L chain. Meanwhile, they also have ferroxidase activity, which can oxidize Fe^2+^ to Fe^3+^ through oxygen and store it in the protein [7,8,102]. However, this does not mean that they are completely identical in terms of function and biochemical characteristics (Table 1).

**Table 1:** Comparison of molecular characteristics between FtMt and FtH.

Characteristic	FtMt	FtH
Subcellular localization	mitochondria	cytoplasm
Structural	24 homologous polypeptides form a cage-like structure	The 24 H chains and L chains are combined in different proportions to form heteropolymers that constitute a cage-like structure.
Ferroxidase activity	The activity of ferroxidase is low, and the oxidation efficiency of Fe^2+^ is slower, resulting in the formation of intermediate state MVFC. At this time, only half of Fe^2+^ is oxidized, and completing two Fe^2+^ requires two O_2_.	Heavy chains have strong ferroxidase activity, while light chains have no enzymatic activity but a stable structure; Fe^2+^ oxidation efficiency is higher, and completing the oxidation of two Fe^2+^ requires only one O_2_ without any intermediate state generation
Tissue expression	The basic expression level is low. The specific expression is highly concentrated in metabolically active or highly oxidative stress tissues such as the brain, heart, and testis.	It is widely expressed throughout the body, and its content is extremely high in iron-storing organs such as the liver, spleen, and bone marrow.
Physiological function	1. Maintain the iron homeostasis of mitochondria and ensure the related physiological processes of mitochondria; 2. Isolate free iron in mitochondria, reduce the generation of ROS, and protect the function of mitochondria; 3. Participate in the synthesis of heme and Fe–S clusters, and support the energy metabolism of mitochondria.	1 The main iron storage reservoir in cells regulates the homeostasis of cytoplasmic LIP; 2. Regulating iron absorption and transport
Regulatory mechanism	Regulated by oxidative stress and epigenetic modifications, the related transcription factors are involved in the gene expression process, which does not rely on the IRE/IRP system.	Mainly regulated by cellular iron levels. The 5′ end of FtH mRNA contains IRE. The translation level is strictly regulated by the IRE/IRP system.

Compared to FtH, FtMt is considered to have lower ferroxidease activity [7,11,102], which is thought to be related to the fact that the serine residue at position 144 and only half of the ferroxidase center is functional. However, recent findings challenged this view, indicating that the reduced Fe^2+^ oxidation efficiency of FtMt is not due to partial activity of its ferroxidase center, but rather to its distinct catalytic mechanism [103]. Specifically, FtMt requires two O_2_ to complete the oxidation of Fe^2+^ to Fe^3+^, whereas FtH only requires one. Consequently, under the same oxygen conditions, only half of the Fe^2+^ in FtMt is oxidized. When the first oxygen molecule binds to the Fe^2+^ at the ferroxidase center of FtMt, an unique Fe^2+^/Fe^3+^ mixed-valent ferroxidase center (MVFC) is generated and only 50% of the Fe^2+^ was oxidized. MVFC in the intermediate state will quickly combine with O_2_ to complete the oxidation of the second Fe^2+^. This phenomenon was first discovered in the unique oxidation mechanism of ferritin from a coastal-dwelling cyanobacterium. Researchers found that the tyrosine residue at position 40 is involved in the formation of MVFC in its ferritin, and this site corresponds to the tyrosine residue at position 34 in FtMt [104,105]. This study better explains why FtMt has lower Fe^2+^ oxidation efficiency and determines the key role of tyrosine in it.

Although FtMt is structurally and functionally similar to the cytoplasmic FtH, it differs significantly in physiological function and distribution. Interestingly, FtMt is not expressed widely throughout the body, but rather is mainly concentrated in tissues with high metabolic activity, while being nearly absent in major iron storage organs [7,12]. Meanwhile, considering the special localization of FtMt on mitochondria and the mRNA of FtMt does not contain an IRE sequence, which means that iron changes and IRP cannot regulate its expression. These endow FtMt with unique functions that distinguish it from FtH. Considering these two points together, FtMt is not only related to the metabolism of cellular iron, but also plays an important role in cellular metabolism and mitochondrial respiration. FtMt provides important guarantees for the synthesis of Fe–S clusters and heme by precisely regulating mitochondrial free iron concentration, which has been confirmed in frataxin-deficient yeast and I/R injury [56]. Moreover, FtMt enhances mitochondrial-specific oxidative stress defense. Both FtMt and FtH are involved in cellular iron regulation and play a role in oxidative damage. Given that mitochondria are the primary cellular source of ROS and central to apoptosis pathways, the protective effect of FtMt is especially vital in high-energy-demand cells such as neurons and cardiomyocytes. Thus, in conditions involving mitochondrial iron deposition or energy metabolism-related dysfunction, FtMt plays a unique protective role.

Unlike ferritin, TfR, ferroportin-1 (Fpn1) contain IRE that are influenced by intracellular iron levels and strictly regulated through the IRE/IRP system. In contrast, the genome of FtMt lacks IRE upstream of the coding region, indicating that the expression of FtMt is not affected or regulated by iron status [9]. Instead, FtMt is upregulated in response to oxidative stress, representing another significant difference compared with FtH. Oxidative stress activates transcription factors such as Nrf2, which in turn can drive the transcription of the FtMt gene. Nrf2 is an important transcription factor for antioxidant stress and can be activated in response to ROS. Studies have shown that overexpression of Nrf2 can significantly increase the expression of FtMt [106]. However, it is not clear whether Nrf2 is independent.

Despite growing research, further comparative studies are needed to investigate the differences between Ferritin and FtMt. Both proteins store iron and evidence indicates that FtMt expression can modulate iron availability and alleviate oxidative damage. A pertinent question is whether cytosolic ferritin overexpression yields similar protective effects. Indeed, both proteins can sequester iron, and in certain contexts, overexpression of cytoplasmic ferritin can indeed reduce cellular LIP, alleviate oxidative damage, and have antioxidant effects [107]. Therefore, in terms of functionality, the two have some similar effects partly, but there are still differences in some key factors. The efficacy of FtMt appears more dependent on cell type and stress conditions. In cells with high mitochondrial ROS production, such as cardiomyocytes and neurons, the antioxidant effect of FtMt is more significant than ferritin. Conversely, in conditions of cytoplasmic iron overload, as seen in hereditary hemochromatosis, ferritin plays a more dominant role.

Furthermore, although FtMt is located in mitochondria, which are highly sensitive to respiratory metabolism and ROS generation. However, how FtMt can sense ROS production and up-regulation remains unclear. Potential pathways include transcription activation and post-translational modifications mediated by oxidative stress responsive transcription factors. Future research should focus on comparative studies and analysis of the differences between the two, providing new insights into the role of iron metabolism in human diseases and promoting the development of novel treatment strategies.

## 5 Mitochondrial ferritin dictates cellular fate by modulating mitochondrial redox balance

Over the years, accumulating evidence has revealed that apoptosis is not the sole form of regulated cell death. A number of other death programs have emerged including autophagy-dependent cell death, necroptosis, ferroptosis and have been reported to be involved in processes of a variety of diseases. Although each of these processes exhibits distinct morphological and mechanistic features, mitochondria plays a central role in nearly all kinds of cell death pathways. Mitochondrial redox imbalance is a key contributor to multiple forms of cell death, highlighting the importance of maintaining cellular redox homeostasis for determining cell fate. Numerous studies have demonstrated that mitochondrial ferritin significantly contributes to cellular antioxidant defense mechanisms. Overexpression of mitochondrial ferritin has been shown to attenuate oxidative damage across various neurodegenerative disease models, highlighting its protective effect in cellular survival.

### 5.1 Mitochondrial ferritin and apoptosis

FtMt has been demonstrated to mitigate oxidative stress and suppress apoptosis by regulating intracellular free iron levels across multiple disease models. Many published articles have shown that FtMt inhibit ROS induced by various toxic oxidizing reagent through regulating cellular iron distribution and availability. For instance, upon direct treatment with H_2_O_2_ and antimycin A, overexpression of FtMt reduced the release of cytochrome *c*, thereby attenuating cellular oxidative damage and apoptosis [10,56]. In an AD model using Aβ-treated rat hippocampus, siRNA-mediated knockdown of FtMt significantly increased cell apoptosis and Cyt *c* release, accompanied by elevated malondialdehyde (MDA) and oxidative injury [13]. Further investigations revealed that this was associated with phosphorylated and activation of p38 and Erk within the MAPK signaling pathway, which triggered apoptosis signals, leading to a decrease in the Bcl-2/Bax and apoptosis [13]. The same effect was also found in mouse models [108]. This process is mainly completed by mitochondrial independent signaling pathways. Therefore, reduction of cytoplasmic iron levels and ROS represents a critical mechanism for alleviating apoptosis. The excessive accumulation of iron in LIP promotes ROS generation, which is an important source of cellular oxidative stress and apoptosis. Overexpression of FtMt regulates cellular iron distribution [52], alters the expression of iron-related proteins Fpn1, TfR1, and DMT1, lowers cytoplasmic LIP levels, and ultimately reduces oxidative stress and apoptosis [13]. Similarly, overexpression of FtMt markedly decreased apoptosis following MPTP and H_2_O_2_ treatment [15,49]. Both MPTP and H_2_O_2_ are established inducers of oxidative damage that upregulate the expression of FtMt and promote apoptosis, underscoring the protective role of FtMt through iron chelation and ROS reduction. Previous studies have shown that under treatment with MPTP and H_2_O_2_, the expression of iron uptake protein TfR1 increased and the expression of iron release protein Fpn1 decreased, leading to an increase in LIP levels in the cytoplasm, resulting in excessive iron accumulation and subsequent ROS production. After overexpression of FtMt in cells, excess iron in the cytoplasm enters FtMt and is isolated, preventing the cytotoxicity and oxidative damage caused by excessive iron, alleviating apoptosis after treatment with various oxidative reagent [15,49,50]. Additionally, FtMt can inhibit the membrane potential decrease caused by MPTP and protect mitochondrial integrity. This result suggests that FtMt not only plays a role in mitochondrial independent apoptosis pathways, but may also be involved in mitochondrial dependentapoptosis. By regulating mitochondrial LIP, FtMt attenuates intra-dependent ROS generation, preserves mitochondrial membrane integrity, and prevents apoptosis by isolating free iron in mitochondria. Treatments such as Aβ, H_2_O_2_, and hypoxia markedly elevate cellular iron levels, whereas overexpression of FtMt mitigates oxidative damage and suppresses ROS mediated apoptosis [96].

Overexpression of FtMt reduces iron imbalance in brain microvascular endothelial cells, decreases oxidative damage, and apoptosis in ischemia/reperfusion (I/R) injury [55]. Collectively, these studies demonstrate that FtMt modulates the distribution of cellular iron, reduces the iron content of cytoplasm and mitochondrial LIP, limits iron availability, and counteracts iron deposition-induced oxidative stress and ROS production [10], thereby exerting a protective effect across diverse models of oxidative damage. While most studies have focused on the role of FtMt in iron redistribution and antioxidative metabolism, one study examining I/R injury revealed its function in apoptosis and antioxidant activity from a mitochondrial bioenergetics perspective. In both in vivo and in vitro I/R models, overexpression of FtMt reduces endoplasmic reticulum stress (ER stress)-induced apoptosis, enhanced maximal respiratory capacity, and increased glycolytic reserve. By promoting the pentose phosphate pathway, FtMt boosted production of NADPH and GSH, thereby diminishing ROS generation and apoptosis after I/R injury [109].

### 5.2 Mitochondrial ferritin and ferroptosis

As the name implies, ferroptosis is a form of iron dependent regulated cell death that was first discovered and defined in 2012 by Dixon et al. in human fibrosarcoma cells [46]. It is distinct from other biological processes such as apoptosis, autophagy, and necrosis in both morphological and biochemical aspects [46]. Key features of ferroptosis include iron-dependent accumulation of ROS [46]. In contrast to classical forms of cell death, ferroptosis is characterized by significant mitochondrial wrinkling and reduction in volume [110,111]. Treatment with Erastin leads to a gradual decrease in mitochondrial cristae over time, accompanied by mitochondrial depolarization and loss of mitochondrial membrane potential [112], indicating impaired mitochondrial function. Although numerous studies have focused on ferroptosis, its precise molecular mechanism remains incompletely understood due to the involvement of multiple signaling pathways [113].

Iron plays a central role in ferroptosis, and dysregulation of iron metabolism directly influences cellular susceptibility to this process. Cellular iron homeostasis is tightly regulated through mechanisms involving iron uptake, storage, and export. Thus, modulating intracellular iron distribution or chelating excess free iron in the cytoplasm can reduce sensitivity to ferroptosis. Ferritinophagy mediated by NCOA4 increases the free iron content in cells, promoting sensitivity to lipid oxidative damage and ferroptosis [97,114]. Consequently, depleting free iron or inhibiting ferritinophagy can attenuate ferroptosis sensitivity.

Accumulating evidence indicates that FtMt plays an important role in mitigating cellular oxidative damage and ferroptosis. Direct treatment of SH-SY5Y cells with H_2_O_2_ resulted in the overexpression of FtMt, which was associated with increased cell survival rate, decreased free iron levels, and reduced cellular oxidative damage [49,50]. Ferroptosis has been identified as the primary mode of oxidative stress-induced cell death in CM, rather than apoptosis or necrosis [115]. Wang et al. demonstrated in *Drosophlia* that ferroptosis inducer Erastin significantly reduced survival, whereas overexpression of FtMt rescued cell death caused by Erastin [116]. Consistent results were confirmed in FtMt-overexpressing SH-SY5Y cells [116]. Further investigation revealed that FtMt regulates cellular iron metabolism, reduces cytoplasmic free iron and ROS levels, and suppresses ferroptosis. Meanwhile, FtMt reduces VDACs and NOXs on the outer membrane of mitochondria induced by Erastin, enhancing mitochondrial stability [116]. Studies on mouse I/R injury revealed that FtMt-knockout mice showed higher levels of LIP, lipid peroxidation, and neuronal iron accumulation following I/R^51^. The deficiency of FtMt exacerbates the inflammatory response after I/R injury, and Hepcidin expression is significantly upregulated, promoting the internalization and degradation of Fpn1 in neurons, preventing iron output after I/R, leading to abnormal accumulation of iron in cells, and ultimately triggering ferroptosis [51]. Furthermore, the study on type 2 diabetes-induced osteoporosis (T2DOP) further revealed the role of FtMt in ferroptosis. T2DOP enhanced both ferroptosis and FtMt expression in rat osteoblasts, while downregulation of FtMt increased high glucose induced ferroptosis in osteoblasts, along with enhanced mitophagy [117]. These findings suggest a potential interplay between mitophagy and ferroptosis. Consistent with this, the mitophagy agonist CCCP promoted ROS production and ferroptosis [117]. In addition to mitophagy, autophagy also participates in the process of ferroptosis. Erastin treatment significantly upregulated autophagy levels in hippocampal HT22 neurons, while the use of autophagy inhibitor 3-MA significantly rescued ferroptosis induced by Erastin [118]. Published studies indicate that ferroptosis is not an isolated process but is closely associated with other modes of cell death such as apoptosis, autophagy, and mitophagy. FtMt appears to be involved in regulating these inter-connected pathways.

### 5.3 Mitochondrial ferritin and other forms of cell death

In addition to apoptosis and ferroptosis, FtMt is also involved in other cell death processes. However, to date, no evidence has established a link between FtMt and either necrosis or pyroptosis. Studies have shown that FtMt is involved in autophagy, mitophagy [92,99,119].

Research on the relationship between autophagy and FtMt remains limited. The most direct evidence comes from a 2014 study on Doxorubicin (DOX) induced cardiotoxicity [120]. In this study, DOX was used to induced acute heart injury, resulting in death in nearly all FtMt-knockout mice, whereas wild-type mice showed a mortality rate of only 60%. FtMt-knockout mice exhibited enhanced oxidative stress damage following DOX treatment, although apoptosis remained unaffected. On the contrary, levels of the autophagy-related protein LC3B-II were significantly elevated in FtMt-knockout mice, and further increased upon DOX treatment. These findings suggest that FtMt may be involved in mediating autophagy-related processes within cells. In addition, FtMt accumulation and colocalization with LC3 were also found in the substantia nigra of patients with progressive nuclear paralysis [119], indicating that FtMt is not only associated with autophagy but also contribute to the pathological mechanisms of this disorder.

NCOA4 is a key regulatory molecule that selectively mediates ferritinophagy [121], it not only participates in ferritinophagy, but also mediates mitophagy. In human primary macrophages, both hypoxia alone and knockdown of NCOA4 significantly upregulated FtMt expression. Combined treatment of hypoxia and NCOA4 knockdown further enhanced FtMt protein, but not RNA levels, supporting a functional link between FtMt protein expression and NCOA4 [97]. The study by Hara et al. further substantiated the relationship between NCOA4 and FtMt [92]. The specific regulation mechanism was proved to the direct binding of proteins and enters the lysosome to degrade. FtMt is involved in iron loss-induced mitophagy and a direct interaction between the two was determined through GST pull-down [92]. This interaction was additionally confirmed through immunofluorescence colocalization, providing direct evidence that FtMt interacts with NCOA4 in the regulation of mitophagy.

### 5.4 The central role of FtMt in various cell death

**Fig. 4:**
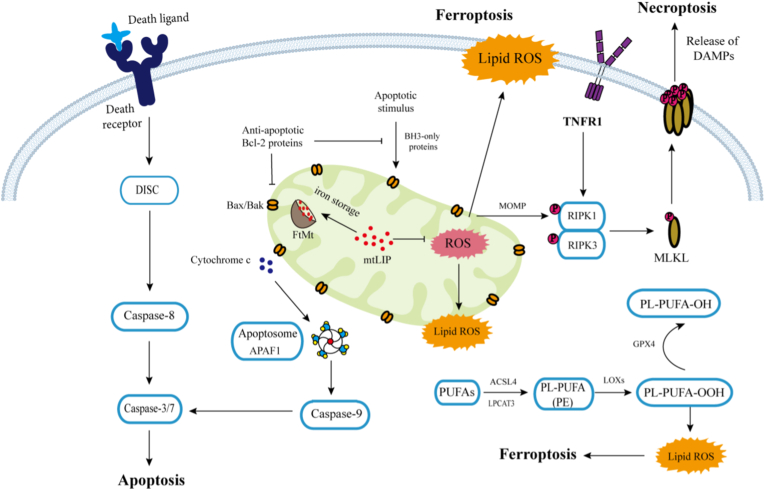
Mitochondria are involved in mediating various cell death mechanisms. Mitochondria-mediated intrinsic apoptosis and receptor-mediated extrinsic apoptosis are the primary pathways of cell apoptosis. Death receptors such as CD95 and Fas bind to and respond to death ligand signals, forming the death-inducing signaling complex (DISC). This recruits and activates caspase-8, which in turn activates downstream caspases-3/7, leading to the cleavage of substrate proteins and ultimately causing cell apoptosis. Intrinsic apoptotic signals trigger the pro-apoptotic proteins Bax/Bak to form pores in the outer mitochondrial membrane, allowing cytochrome *c* to be released into the cytoplasm. Cytochrome *c* then binds to APAF1 to form the apoptosome, which activates downstream caspase-9 and caspase-3, initiating apoptosis. TNF-α or changes in mitochondrial outer membrane permeability can trigger cell necrosis. RIP1 and RIP3 form a complex, which phosphorylates and activates MLKL, causing it to oligomerize and insert into the cell membrane, forming pores that lead to membrane rupture. Mitochondrial iron overload promotes the production of ROS through the Fenton reaction. This process further amplifies intracellular cascades, generating lipid ROS and triggering ferroptosis.

Mitochondrial ferritin, as a key iron metabolism regulatory protein located in mitochondria, not only plays a role in iron ion storage and maintaining iron homeostasis, but also acts as a core hub and arbitrator in the precise regulatory network of programmed cell death (PCD). FtMt regulates mitochondrial redox through its unique iron sequestration function and participates in the regulation of various cell death pathways directly or indirectly (Fig. 4). By sequestering excess iron in its cage-like structure within mitochondria, FtMt reduces excessive free iron in mitochondria, inhibits ROS generated by the Fenton reaction, and thereby alleviates mitochondrial oxidative damage, ultimately suppressing ferroptosis and apoptosis via the mitochondrial pathway. Moreover, FtMt can also regulate cytoplasmic LIP, facilitating the preferential entry of iron into mitochondria and storage in FtMt, which further inhibits iron-dependent lipid peroxidation and excessive ROS production, as well as the subsequent ferroptosis. ROS serves as a critical signal for activating mitophagy. While moderate ROS level promote mitophagy to remove damaged mitochondria and maintain mitochondrial homeostasis, excessive ROS may lead to uncontrolled autophagy and even shift towards apoptosis or ferroptosis. Thus, by centering on mitochondrial iron homeostasis and ROS control, FtMt orchestrates cell-fate decisions through interconnected molecular mechanisms. Rather than merely affecting a single pathway, FtMt profoundly integrates the apoptosis, ferroptosis, and autophagy/mitophagy networks by sustaining a common foundation of mitochondrial iron and redox balance (Table 2).

**Table 2:** The role of FtMt in different cell death.

Cell Death	Mechanisms	Key molecular	Related Disease	Reference
Apotosis	Reduce the generation of mitochondrial ROS, mitigate damage to mitochondrial membrane potential, and inhibit the activation of the caspase pathway; Maintain mitochondrial iron homeostasis and avoid apoptosis signals induced by iron overload;	Caspase-3、Bcl-2/Bax、mtROS、cytochrome C	Alzheimer's disease; Parkinson's disease; Ischemia/Reperfusion	13, 15, 50, 51, 55, 96, 108, 109
Ferroptosis	Storing free iron in mitochondria, reducing the substrate for the Fenton reaction, and inhibit iron-dependent lipid peroxidation; It protected the functional integrity of mitochondria and maintained the efficacy of antioxidant systems.	ACSL4、iron、ROS、lipid peroxidation	Ischemia/Reperfusion	51, 99, 101, 114-118
Autophagy	It is related to ferroptosis, but the specific mechanism remains unclear. The ferroptosis inducer Erastin significantly upregulates autophagy in cells, while the autophagy inhibitor 3-MA can alleviate the cell death induced by Erastin.	LC3	Acute heart injury;	118, 119, 120
Mitophagy	NCOA4 directly binds to FtMt and further recognizes LC3, encapsulating the damaged mitochondria into autophagosomes and degrading them.	NCOA4、LC3b	Type 2 diabetes-induced osteoporosis	92, 97, 99, 117

## 6 Mitochondrial ferritin and related diseases

**Fig. 5:**
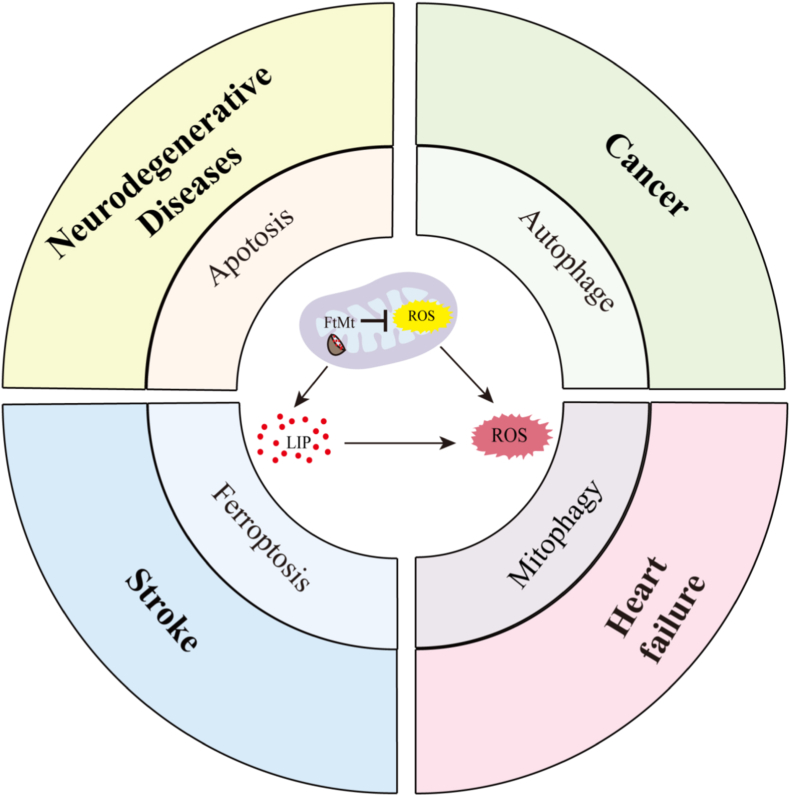
Mitochondrial ferritin plays a protective role in various diseases by regulating intracellular iron metabolism and oxidative stress. The expression of mitochondrial ferritin can isolate iron in mitochondria and also absorb iron in the cytoplasm. On the one hand, this process can reduce the level of LIP in the cytoplasm and the resulting increase in ROS, alleviating ferroptosis. On the other hand, it prevents mitochondrial dysfunction caused by iron overload, which subsequently leads to the release of cytochrome *c* and apoptosis. In addition, the expression of FtMt plays a protective role in cellular autophagy by reducing the changes in LC3B.

The accumulation of intracellular iron increases with age, and iron dysregulation has been closely linked to age-related neurodegenerative diseases such as Alzheimer's disease and Parkinson's disease [122,123]. While iron is essential for multiple important physiological processes, excessive iron accumulation contributes to cellular damage and the pathogenesis of various diseases. In neurodegenerative diseases, iron deposition promotes oxidative stress, leading to loss of neurons and synapses, and ultimately resulting in memory impairment or motor dysfunction [124]. It has been reported that FtMt plays a protective role in various diseases, including neurodegenerative disease, Friedrich's ataxia, tumors, and stroke [13,15,109,125,126](Fig. 5).

### 6.1 The role of mitochondrial ferritin in neurodegenerative diseases

Alzheimer's disease is a common neurodegenerative disease characterized by progressive decline in memory as well as cognitive function [[127], [128], [129]]. The typical pathological features are plaques formed by excessive phosphorylation of Tau protein and abnormal accumulation of Aβ in the brain [[127], [128], [129]]. Dysregulated iron metabolism and oxidative stress are strongly correlated with AD pathogenesis [122]. The accumulation of iron promotes both deposition of Aβ and Tau phosphorylation, exacerbating the pathology of AD [122,130]. Multiple iron-regulating proteins have been reported to involve in the pathological process of AD [[131], [132], [133]]. There are researches report that FtMt located in mitochondria affect the pathological process of AD due to its important role in regulating cellular iron distribution and redox balance, but research on the relationship between FtMt and AD is still very limited. The in situ hybridization results revealed significantly elevated FtMt expression in the cerebral cortex of AD patients, with increased number and staining intensity of positive neurons [134]. Real-time PCR and immunoblotting experiments also supported this result, confirming that FtMt is expressed in the temporal lobe cortex of AD patients rather than the cerebellum [134]. Further validation in IMR-32 cells showed that H_2_O_2_ treatment significantly upregulated FtMt expression, while Aβ treatment alone had no effect. Combined treatment with both H_2_O_2_ and Aβ further enhanced FtMt expression [134]. Overexpression of FtMt attenuated H_2_O_2_-induced cytotoxicity and improved cell viability. These results suggest that Aβ may exert toxic effects through oxidative stress, and FtMt is significantly upregulated in response to intracellular oxidative stress, thereby participating in neuroprotective effects against cell toxicity. Wu et al. further investigated the neurotoxic mechanism of FtMt in protecting Aβ_25-35_-induced neurotoxicity in rats and SH-SY5Y cells [13]. Overexpression of FtMt in SH-SY5Y cells mitigated Aβ-related toxic effects. The same phenomenon results were observed when Aβ_25-35_ was injected into the lateral ventricle of WT and FtMt-knockout mice [108].

The loss of dopaminergic neurons in the substantia nigra pars compacta is an important characteristic leading to the onset of PD [135]. The Lewy bodies formed by the aggregation of α-synuclein are the main substances responsible for neurotoxicity in PD [136]. The accumulation of iron in the substantia nigra pars compacta, oxidative stress, and mitochondrial dysfunction are closely involved in the pathogenesis of PD [137,138]. Although the role of iron in PD has been extensively studied, reports on FtMt in PD remain relatively limited. Tsubaki et al. investigated the localization and expression of FtMt in the midbrain of PD patients. FtMt was detected in both healthy individuals and PD patients, mainly concentrated in the compact part of substantia nigra. Notably, the FtMt levels were significantly elevated in PD patients compared to control, indicating a correlation between FtMt and PD [139]. Previous studies have shown that FtMt colocalizes with approximately 70% of neurons in the dense area of the substantia nigra, implying a role in its physiological functions [90]. To further investigate the relationship between FtMt and PD, a stable FtMt-transfected SH-SY5Y cell line was established. Overexpression of FtMt significantly rescued the decrease in cell viability caused by 6-hydroxydopamine (6-OHDA), and significantly attenuated the expression of apoptosis-related proteins, including Bcl2, Bax, and Caspase. Mechanistically, FtMt increases iron release after 6-OHDA treatment, lowered cytoplasmic LIP levels, and decreases ROS production via suppression of iron-mediated Fenton reactions, thereby exerting neuroprotective effects [14]. This result was further validated in mice. MPTP injection significantly upregulated the FtMt in the hippocampus, substantia nigra, and striatum of mice. FtMt-knockout mice exhibited enhanced apoptosis, iron deposition, and increased ROS levels after MPTP treatment [15]. Collectively, these results demonstrate a protective role of FtMt in PD pathology. Further investigation into the interplay between iron and α-synuclein revealed additional insights into FtMt's function. Guan et al. demonstrated that FtMt modulates α-synuclein expression in SH-SY5Y cell. The iron chelator DFO inhibited the expression of α-synuclein, and overexpression of FtMt produced a similar effect, which was attributed to reduced intracellular LIP levels. On the contrary, there were higher LIP and α-synuclein levels after FeCl_2_ treatment. These results demonstrate that FtMt regulates α-synuclein expression by modulating the availability of iron and reducing the level of LIP in cytoplasm.

FRDA is a severe genetic degenerative disorder affecting both the heart and nerves system. A major causative factor is mutation or deficiency in the frataxin gene, which is localized in mitochondria. Frataxin is involved in the synthesis of mitochondrial Fe–S clusters and heme iron. This gene defect can lead to mitochondrial iron overload, increased oxidative toxicity damage to mitochondria, and ultimately result in cell death and tissue damage. The significant upregulation of FtMt was found in the hearts of FRDA patients, suggesting a close association between FtMt and FRDA. FtMt plays a role in iron storage and reducing oxidative damage in mitochondria, indicating upregulation of FtMt may represent a compensatory response in FRDA. Constructing cells expressing human FtMt in frataxin-deficient yeast, it was found that frataxin-deficient yeast hardly grows or grows slowly on a carbon source, while cells expressing FtMt can grow normally on a carbon source, but partial deletion of the FtMt gene restores the phenotype of frataxin-deficient yeast [56]. Furthermore, FtMt expression partially recovered intracellular Fe–S enzyme activity and increases cell resistance to H_2_O_2_. The experiment in HeLa cells also confirmed this conclusion [57]. Similarly, expression of FtMt in fibroblasts derived from FRDA patients reduced cellular ROS levels and increased aconitase activity, leading to improved cell viability [10].

### 6.2 The role of mitochondrial ferritin in stroke

Stroke is currently a very common cerebrovascular disease, divided into hemorrhagic stroke and ischemic stroke. Due to various factors such as hypertension, blood vessels rupture, causing blood to flow out and accumulate in the brain, which in turn compresses brain tissue, known as hemorrhagic stroke [140,141]. And vascular embolism leading to blood flow interruption, which in turn causes brain tissue necrosis, is called ischemic stroke, which is also the most common mode of stroke [141]. Iron has always been considered closely related to stroke. Research has shown that iron deposition is observed on the injured side of stroke, and this phenomenon contributes to stroke [142]. Although the specific molecular mechanism of iron deposition on the injured side after ischemia is still unclear. Ding et al. found that after I/R injury, the expression of ferritin in the cortex of mice on the injured side significantly increased, while Fpn1 decreased [143]. This process was accompanied by a significant increase in Hepcidin and HIF-1α, which is considered an important cause of iron deposition after ischemic stroke [143]. The use of iron chelators DFO significantly alleviates stroke damage [144]. FtMt has been proven to exhibit excellent protective effects in stroke injury due to its specific role in cellular iron regulation and redox processes. Initially, FtMt was found to significantly upregulate in response to ischemia. FtMt-deficient mice showed higher iron levels, lipid peroxidation, and ferroptosis after I/R injury [51]. Moreover, increased apoptosis was detected in FtMt-deficient mice after I/R injury [109]. Further studies revealed that FtMt-overexpressed SH-SY5Y cells exhibited higher mitochondrial respiration and glycolytic capacity, with significant increases in the pentose phosphate pathway and NADPH, thereby enhancing the cells’ ability to resist I/R-induced apoptosis and oxidative damage [109].

In addition, the effect of FtMt overexpression on mitochondrial respiration of retinal pigment epithelial cells was detected by Seahorse, and it was found that overexpression of FtMt decreased basal respiration and maximal respiration volume, but did not affect total ATP production [145]. This finding appears to contrast with studies in ischemic stroke model, where overexpressing FtMt enhanced both respiration and glycolytic capacity after OGD/R, enhancing the pentose phosphate pathway to produce NADPH [109]. Consistent with this, reseatch by Chang et al. in hypoxia-induced ferroptosis of neonatal rat ventricular myocytes similarly indicated that FtMt can modulate cell survival by affecting mitochondrial bioenergetics [146]. These results provide evidence for the impact of FtMt on mitochondrial function and respiratory processes. However, the precise mechanism by which FtMt overexpression enhances cellular respiratory processes remains to be fully elucidated. One proposed pathway involves the role of FtMt in regulating mitochondrial heme and Fe–S cluster synthesis. By modulating the mitochondrial LIP, FtMt may indirectly support the production of heme and Fe–S cluster, both of which are essential cofactors for key enzymes in cellular metabolism and respiration—such as aconitase (which shares structural homology with IRP) and components of the mitochondrial electron transport chain. Thus, through its effect on these essential iron-containing complexes, FtMt may exert a broader influence on cellular respiration and energy metabolism.

FtMt also plays an important role in cerebral hemorrhage. Yuan et al. conducted research on Germinal Matrix Hemorrhage (GMH) and elucidated the crucial role of FtMt in it. GMH can significantly upregulate the expression of FtMt. At the same time, iron deposition occurs in endothelial cells of rat pups, and the blood-brain barrier (BBB) is damaged [126]. Administration of the iron chelator Deferiprone (DFP) significantly improves iron deposition and ferroptosis caused by GMH, and this effect is eliminated by the knockout of FtMt [126]. That is, compared with the DFP treatment group, the combined treatment of DFP and FtMt knockout results in greater BBB damage and neurobehavioral deficits in rat pups [126]. On the contrary, overexpression of FtMt through adenovirus alleviates oxidative stress and BBB damage caused by FeCl_2_, although the authors used FeCl_2_ to simulate iron deposition rather than the GMH injury model [126]. These results provide direct evidence for the important role of FtMt in regulating cellular free iron, reducing oxidative stress and cell death in cerebral hemorrhage and ischemic stroke (Table 3).

**Table 3:** The mechanism of action of FtMt in different diseases.

Disease Type	Mechanisms	Cell Death Type	Reference
Alzheimer's disease	1. Alter the iron homeostasis of cells and inhibit the oxidative damage caused by Aβ; 2. Reduce ROS production, maintain stable mitochondrial membrane potential, inhibit the release of cytochrome *c* and cell apoptosis; 3. Alleviate the memory impairment in mice induced by Aβ	Apotosis	13, 55, 108, 134
Parkinson's disease	1. Reduce the iron content of dopaminergic neurons and inhibit the aggregation of alpha synuclein; 2. Maintain mitochondrial homeostasis, reduce mtROS and cell death; 3. Reduce the level of free iron in the cytoplasm and decrease the generation of reactive oxygen species (ROS) by inhibiting the iron-mediated Fenton reaction	Apotosis	14, 15,
FRDA	FtMt regulates mitochondrial iron levels, promotes Fe–S enzyme activity, and enhances mitochondrial respiration and energy generation	—	10, 56, 57
Ischemia/Reperfusion	1. Chelate free iron in cells, maintain mitochondrial iron homeostasis, reduce ROS production after I/R, and inhibit apoptosis and ferroptosis; 2. Enhance and protect mitochondrial respiration, maintain ATP production; 3. The pentose phosphate pathway is enhanced, NADPH significantly increases, increasing the reductive capacity of cells and their resistance to oxidative damage.	Apotosis; Ferroptosis	51, 55, 109, 143
Germinal Matrix Hemorrhage	Isolate free iron and reduce oxidative stress, alleviating iron overload and ferroptosis caused by GMH	Ferroptosis	126
Heart failure	In cardiac injury caused by DOX、tBHP、I/R treatment, expression of FtMt in cardiomyocytes can alleviate mitochondrial iron deposition and reactive oxygen species (ROS) caused by different treatments and alleviate cell death	Autophagy; Ferroptosis	115, 150, 151, 152, 153, 154
Acute exhaustive exercise	Regulate the free iron level in cells, maintain mitochondrial homeostasis, and inhibit apoptosis caused by AEE	Apotosis	16
Epilepsy	Regulate cytoplasmic and mitochondrial LIP, inhibit ROS and lipid peroxidation, and alleviate neuronal ferroptosis induced by KA	Ferroptosis	106
Cancer	Dual roles: 1. Promotes survival: As a mitochondrial iron storage reservoir, it meets the high metabolic demands; inhibits the production of ROS, helping tumors tolerate the hypoxic microenvironment and resist ferroptosis. 2. In specific situations, chelates the iron required for cell growth and differentiation, halts the cell cycle, and inhibits tumor growth.	—	93, 125, 161, 162

### 6.3 The role of mitochondrial ferritin in heart disease

Similar to the brain, the heart is a high-energy and high-metabolic organ responsible for transporting blood throughout the body, and requires more iron to maintain normal physiological functions. FtMt is predominantly expressed in cells with high energy requirements, such as those in the heart and brain. Therefore, FtMt is not only associated with neurological diseases, but also plays an important role in heart diseases. Iron homeostasis is essential for maintaining normal cardiac physiology, while iron imbalance can lead to heart disease [147,148]. Numerous studies have established that both iron overload and iron deficiency are associated with cardiac pathologies [149,150].

In recent years, increasing attention has been directed toward understanding the relationship between FtMt and heart disease. Early research on FtMt in heart disease focused on DOX-induced cardiotoxicity, although HeLa cells were also used in the study [151]. DOX, as an anti-cancer drug, can produce cardiotoxic side effects during cancer treatment, which are thought to arise from iron homeostasis imbalance. After DOX treatment, ferritin in the cardiac tissue of mice significantly increased, whereas the FtMt mRNA expression remained unchanged. However, overexpression of FtMt in HeLa cells markedly attenuated DOX-induced iron accumulation and loss of cell viability, while also enhancing cell sensitivity to H_2_O_2_ and antioxidant capacity [151]. Considering the high expression of FtMt in the heart and muscles, acute exhaustive exercise (AEE) was used to detect the role of FtMt in heart and related oxidative damage, which is a common oxidative stress model [16]. The results showed that FtMt significantly increased after AEE, and the hearts in FtMt-knockout mice had higher levels of cell apoptosis and oxidative stress after AEE. This result is attributed to the significant increase in calcium and free iron levels in cardiac slice tissue detected by synchrotron radiation X-ray fluorescence (SR-XRF) [16]. After AEE, the levels of calcium and free iron in mouse heart tissue slices increased, and FtMt knockout exacerbated this phenomenon, which better explains why FtMt-knockout mice have higher apoptosis and oxidative stress after AEE [16]. Calcium activation is involved in various signal transduction and physiological processes within cells, including apoptosis, while more free iron in the cytoplasm generates ROS through the Fenton reaction, which may further exacerbate apoptosis. Therefore, FtMt-knockout mice are more prone to AEE-induced oxidative damage and cell death due to iron metabolism imbalance.

Chen et al. isolated primary cardiomyocytes from rat heart tissue and demonstrated the protective role of FtMt against various stress stimuli. In models of oxidant-induced cell death using *tert*-butyl hydroperoxide (tBHP), tBHP promoted mitochondrial iron accumulation and lipid peroxidation. Overexpression of FtMt in cardiomyocytes mitigated mitochondrial iron deposition and ROS caused by tBHP or DOX, IR, thereby reducing cell death [115,150,152]. These findings confirm that FtMt exerts antioxidant and protective effects in cardiomyocytes. In a model of isoproterenol induced heart failure, IRP levels were significantly decreased in mouse cardiomyocytes, along with decreased of the iron input protein TfR1 and output protein Fpn1 [153]. The expression of FtMt was also downregulated, and total iron levels were significantly reduced, although cytoplasmic ferritin remained unchanged [153]. This result seems to be inconsistent with the classical IRP/IRE regulatory mechanism, suggesting the existence of other non classical regulatory mechanisms. Although FtMt lacks an IRE, its expression may be indirectly regulated by transcriptional levels or epigenetic mechanisms. The decrease in IRP levels could influence FtMt transcription by perturbing intracellular iron homeostasis or mitochondrial iron metabolism, thereby activating downstream signaling pathways, such as ROS sensitive pathways and nuclear transcription factor. In addition, mitochondrial iron transporters Mfrn2 also decreased after treatment with isoproterenol, although Mfrn1 showed no significant changes. This result will lead to a decrease in mitochondrial iron supply, resulting in mitochondrial dysfunction and ultimately downregulation of FtMt expression, while cytoplasmic ferritin remains relatively independent and unaffected. These results were supported by a clinical trial, showing that, in patients with heart failure, most iron related proteins were downregulated, and iron storage proteins FtH, FtL, and FtMt were significantly decreased, while the exporter Fpn1 showed no significant change [154]. This indicates decreased iron in the cardiomyocytes of heart failure patients and implies potential role for FtMt in heart failure pathogenesis providing compelling evidence for its association with heart disease. Although altered the expression of FtMt has been observed in heart failure, the precise mechanisms underlying its action and the implications of decreased iron levels are still unclear.

### 6.4 The role of mitochondrial ferritin in epilepsy

Epilepsy, as a common chronic neurological disease, is caused by abnormal discharge of neurons and further uncontrollable physiological behaviors such as seizures due to neuronal damage [155,156]. The pathogenesis of epilepsy is relatively complex, with multiple cell deaths involved, and the understanding of this disease is still limited. Iron, as an essential trace element for the brain, has become an important factor in the onset of epilepsy [157]. There have been research reports that iron deposition and oxidative stress damage occur in the lesion area of epilepsy patients, and the use of iron chelators can significantly alleviate the pathogenesis of epilepsy, suggesting that iron is involved in the pathological occurrence of epilepsy [[158], [159], [160]]. The unique advantages of FtMt in the distribution of iron in cells and its role in antioxidation play an important role in alleviating iron deposition and oxidative damage in epilepsy, although there are few studies on FtMt and epilepsy. A recent study elucidated the role of FtMt in epilepsy. After stereotactic injection of kainic acid (KA) to induce epileptic seizures in mice, there was an increase from day 3 to 28 and a peak was reached on day 7. At the same time, the number of neurons decreased due to ferroptosis, suggesting that FtMt plays a specific role in KA induced epilepsy and ferroptosis [106]. Injecting adeno-associated virus (AAV) into the hippocampus of mice to knock down FtMt exacerbates ROS generation and ferroptosis, while overexpression of FtMt alleviates KA induced behavioral damage and neuronal loss [106].

### 6.5 The role of mitochondrial ferritin in cancer

Cancer is currently a disease characterized by high mortality, broad incidence, and lack of effective treatment. Given the crucial role of iron in cellular division, growth, and metabolism, its importance is even more pronounced in tumor cells, which exhibits accelerated growth, proliferation, and metastasis processes that requires increased iron availability. FtMt influences tumor cell growth and metabolism by regulating cytoplasmic iron levels and cell cycle progression [125]. Initially, Nie et al. investigated the relationship between FtMt and tumors by injecting FtMt-overexpressing H1299 cells subcutaneously into nude mice to explore the effect of FtMt on tumor iron metabolism and growth. After 4 and 6 weeks, changes in iron metabolism in tumor cells were detected, and it was found that overexpression of FtMt significantly reduced the expression of ferritin in tumor tissue, as well as the lower activity of malate dehydrogenase and the expression of frataxin [161]. The observation results of transmission electron microscopy showed direct mitochondrial iron deposition in FtMt overexpressing cells. This process affects the growth rate of FtMt overexpressing cells, resulting in tumors with lower growth rates and ultimately smaller volumes [161]. This study confirmed that FtMt expression reduces cytoplasmic iron and inhibits tumor growth. Consistently, Shi et al. found that FtMt expression was reduced in glioblastoma and neuroblastoma compared to normal brain tissue [125]. Injection of FtMt-overexpressing SH-SY5Y cells into nude mice led to a significant reduction in tumor volume. Flow cytometry analysis revealed that overexpression of FtMt arrested cells at the G1/S phase without inducing apoptosis, mediated through the downregulation of CyclinD1 and Cdk2, which are important molecules for promoting tumor growth. On the contrary, supplementation with ferric ammonium citrate (FAC) partially reversed iron deficiency and restored cyclin expressionin FtMt-overexpressing cells [125], indicating that FtMt inhibits tumor growth by affecting cellular iron metabolism, which leads to “iron starvation” in cells, resulting in downregulation of cell cycle-related proteins and Rb expression, ultimately leading to cell cycle arrest and slow tumor growth. In addition, FtMt contributes to the Roflumilast-induced suppression of ovarian cancer, and its expression is upregulated via the cAMP/PKA/CREB signaling pathway [93]. Together, these pieces of evidence demonstrate that FtMt and iron inhibit tumor cell proliferation via cell cycle modulation, providing valuable insight for further exploration of FtMt's functional roles. This result seems to contradict the distribution of FtMt in metabolic activity. Interestingly, while some studies support a tumor-suppressive role for FtMt across various cancers, one study reported significant upregulation of FtMt in glioma, and its enhanced expression promoted tumor growth and angiogenesis [162]. This result is completely opposite to previous studies, suggesting that FtMt seems to play a context-dependent roles in tumor.

The dual role of FtMt in tumors highlights a key paradox in current research on tumor metabolism. The “double-edged sword” role of FtMt in tumors precisely reflects that its function in cellular iron homeostasis and redox balance is highly dependent on factors such as tumor type, microenvironment, genetic background, and its own expression level. When FtMt exerts its tumor-suppressing effect, its high expression reduces the levels of labile iron in both mitochondria and the cytoplasm of tumor cells, leading to decreased iron availability and disruption of redox homeostasis. This impairs the ability of tumor cells to utilize elevated levels of ROS and iron demand as proliferative signals, instead inducing cell cycle arrest and senescence, thereby inhibiting tumor growth. In glioma, the oncogenic effect of FtMt may stem from the fact that in the tumor hypoxic core, FtMt expression may be upregulated. FtMt stores free Fe^2+^ within the mitochondria, reducing the free iron available for the Fenton reaction within the mitochondria, thereby decreasing the production of the most destructive •OH. It releases iron when needed to support the rapid proliferation required by tumor cells, without triggering iron toxicity. Moreover, FtMt strongly clears mitochondrial ROS and isolates iron, enabling tumor cells to resist oxidative damage and ferroptosis, thereby achieving greater survival and promoting tumor recurrence and invasion.

FtMt's functional divergence is partly attributed to its expression dynamics. Very high FtMt levels, as in SH-SY5Y overexpression models, can exert a protective and growth-suppressive effect by strongly chelating iron and restricting its availability for proliferation. In contrast, moderate upregulation of FtMt in tumor tissues may sustain energy metabolism and mitigate oxidative damage, aiding adaptation to hypoxic conditions without inhibiting growth. Thus, the net outcome of FtMt activity is shaped not only by its expression level but also by contextual factors including the metabolic state of the tumor and its microenvironment. Future studies should aim to clarify the regulatory signals governing FtMt expression and its context-specific roles across different tumor types. Although research on the anti-tumor mechanism of FtMt has advanced, its full biological significance remains incompletely understood. These studies have elucidated the important role of FtMt in cell growth and tumor suppression. FtMt affects cell cycle and metabolic processes by regulating cytoplasmic iron levels, providing new targets and ideas for future tumor prevention and treatment.

## 7 Conclusion and perspectives

**Fig. 6:**
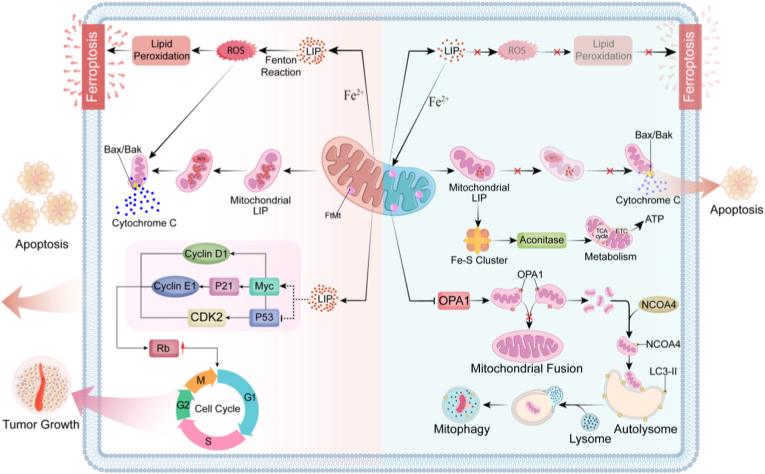
The homeostasis and imbalance of mitochondrial ferritin. Under normal physiological conditions, Fe^2+^ in the cytoplasmic LIP will preferentially enter FtMt, reducing the level of free iron in the cytoplasm and the ROS produced through the Fenton reaction, maintaining the intracellular redox balance, and reducing the susceptibility to lipid peroxidation and ferroptosis. Additionally, FtMt can regulate the mitochondrial LIP to participate in the synthesis of heme and Fe–S clusters in mitochondria, thereby indirectly affecting the normal operation of the electron transport chain and the tricarboxylic acid cycle, promoting cellular energy production. FtMt isolates free iron in mitochondria, maintains mitochondrial redox balance, reduces mitochondrial oxidative damage and subsequent apoptosis. Moreover, FtMt participates in NCOA4-mediated mitophagy. FtMt reduces the expression of OPA1 after hypoxia, inhibits mitochondrial fusion, leading to more damaged mitochondrial fragmentation and degradation through mitophagy. The absence of FtMt affects the expression of cell cycle-related proteins, ultimately resulting in an increase in the key tumor suppressor protein Rb, promoting tumor growth.

This review systematically summarizes the research progress of FtMt over the past two decades. FtMt is specifically located in mitochondria, structurally similar to cytoplasmic ferritin, and can isolate iron and store it in its own cage-like structure. However, the two are not exactly the same in terms of expression regulation and physiological and biochemical characteristics. FtMt regulates the availability of intracellular iron, allowing iron to preferentially enter mitochondria, controlling the levels of free iron and ROS generation in cells, thereby participating in mitochondrial redox regulation and determining cell fate. The specific localization of FtMt in high metabolic organs such as the brain and heart determines that it not only plays a role in iron storage, but also participates in mitochondrial respiration and energy generation. FtMt can effectively inhibit ROS accumulation induced by iron overload and play an important protective role in neurological diseases and cardiovascular diseases [[13], [14], [15],^51^]. The study found that FtMt prevents oxidative damage and cell death caused by iron deposition and maintaining mitochondrial and cytoplasmic iron homeostasis in various diseases. Furthermore, FtMt affects the expression of cell cycle-related proteins by influencing the availability of iron in cells, resulting in a decrease in Rb expression. Eventually, this leads to tumor cell cycle arrest and inhibits tumor growth (Fig. 6).

Although significant progress has been made in the fundamental research of FtMt, there are still unsolved scientific problems in this field. It is worth noting that FtMt does not have iron responsive elements upstream of the coding region, so it is not sensitive to iron. Therefore, an important issue is what signal does the mitochondrion give to activate the transcription of FtMt in response to its demand? Although lacking IRE, the expression of FtMt indeed responds to the cellular iron status in an indirect way. The direct consequence of mitochondrial iron overload is an increase in mitochondrial LIP, which then triggers the Fenton reaction to produce a large amount of ROS. These ROS may act as a “bridge” signal to activate nuclear stress-related transcription factors through the reverse mitochondrial-nuclear signal. Therefore, analyzing the non-classical regulatory network of FtMt is an important direction that needs to be further clarified. In the future, attention should be focused on the non-classical regulatory pathways of FtMt, deepening the mechanism research, screening the intermediate molecules that regulate FtMt, and clarifying its indirect regulatory mechanism. At the same time, further exploration of the interaction between stress pathways such as Nrf2 and NF-κB and the regulation of FtMt should be carried out, and a multi-pathway coordinated regulatory network should be constructed.

Previous research on FtMt mainly focused on iron metabolism and redox balance regulation. Therefore, another important issue that needs attention is the impact of FtMt on the normal metabolic function of mitochondria. Some studies have found that FtMt can participate in NCOA4 mediated ferritinophagy and mitophagy under different stress to increase cell resistance and survival to stimuli [97,99,145], which is another important physiological process of FtMt besides regulating cellular iron metabolism. However, there is limited research on the relationship between FtMt and mitochondria [10,56]. Wang et al. determined the effect of FtMt on mitochondrial fission and fusion in a study exploring the pathophysiology of FtMt and age-related macular degeneration (AMD). Overexpression of FtMt in retinal pigment epithelial cells showed a significant reduction in the mitochondrial fusion protein OPA1, suggesting that FtMt may be involved in mitochondrial quality control [145]. Although the specific molecular mechanism by which FtMt downregulates OPA1 is not yet clear. This study confirms that FtMt is not only a regulator of mitochondrial iron homeostasis, but also may participates in the regulation of mitochondrial morphological dynamic balance. This regulation may ultimately trigger mitophagy, providing key support for understanding the pleiotropic protective effect of FtMt. Apart from the traditional iron binding function, there is currently little research on the function of FtMt. Therefore, in the future, attention should be paid to the non-iron dependent functions of FtMt, such as whether it participates in regulating mitochondrial metabolism and biosynthesis, molecular chaperones, and maintaining mitochondrial DNA stability. At the same time, exploring the cross-border regulatory role of FtMt in different physiological and pathological processes, such as its role in aging, immune response, and metabolic syndrome, expanding the breadth and depth of FtMt research, and exploring its new clinical application value.

FtMt, as a specific iron storage protein located in mitochondria, participates in the occurrence and development of various diseases by regulating mitochondrial iron homeostasis, inhibiting oxidative stress, and other core functions. In recent years, preclinical studies on FtMt have clarified its pathological regulatory role and molecular mechanisms in multiple disease models, laying the foundation for clinical translation. However, there is still a significant lack of data on clinical patients and related cases of FtMt. Currently, most research is based on cell models and animal models. There is a lack of data on the expression of FtMt in human tissues and its changes under different disease states, especially the correlation between FtMt expression and disease progression and prognosis at different disease stages. Large sample clinical cohort studies have not yet been conducted, making it difficult to determine the clinical value of FtMt as a biomarker. In iron-overload related diseases, although there are cases confirming the correlation between FtMt and iron deposition degree, there is a lack of large sample data to clarify the predictive value of FtMt expression level on disease prognosis. In addition, the safe expression range and intervention threshold of FtMt in the human body have not been determined, which poses difficulties for the development of clinical intervention plans.

Preclinical studies have clearly demonstrated the role of FtMt in neurological diseases, cardiovascular diseases, and tumors. Given the significant role of FtMt in the cellular redox balance and cell fate, FtMt may become a new therapeutic target for these diseases. Drugs targeting key high energy consuming organs such as the nervous system, heart, and tumors that induce upregulation of FtMt can be developed to effectively prevent and treat these diseases by controlling cell fate. The core mechanisms all revolve around mitochondrial iron homeostasis regulation, inhibition of oxidative stress, and improvement of energy metabolism, providing a solid theoretical foundation and potential targets for clinical translation. The future development directions should focus on three aspects: First, accelerating the research and development of FtMt-targeted drugs, such as small molecule regulators based on its unique iron oxidation mechanism, and conduct targeted early clinical trials, such as phase I safety assessment and phase II validation of disease control effects; Second, expanding the clinical sample cohort to verify the reliability of FtMt and related iron metabolism molecules as biomarkers for neurodegenerative diseases, cardiovascular diseases, and tumors; Third, exploring the synergistic effects of FtMt with other treatment strategies, providing new ideas for combined treatment regimens. With further research, FtMt is expected to become a new target for diagnosis, prognosis assessment, and treatment of multiple systemic diseases, promoting the development of precision medicine for related diseases.

## CRediT authorship contribution statement

**Yuanyuan Liu:** Writing – original draft. **Yan-Zhong Chang:** Funding acquisition, Writing – review & editing.

## Declaration of competing interest

The authors declare that they have no known competing financial interests or personal relationships that could have appeared to influence the work reported in this paper.

## Data Availability

No data was used for the research described in the article.

## Abbreviations

FtMt: Mitochondrial ferritin
ROS: reactive oxygen species
FtH: ferritin heavy chain
LIP: labile iron pool
AD: Alzheimer's disease
PD: Parkinson's disease
Fe–S: iron-sulfur
Fe^2+^: ferrous iron
Fe^3+^: iron ion
ETC: electron transport chain
TCA: tricarboxylic acid cycle
NADH: nicotinamide adenine dinucleotide
FADH2: flavin adenine dinucleotide
CoQ: coenzyme Q
NADPH: Nicotinamide Adenine Dinucleotide Phosphate
NFS1: Nitrogen Fixation 1-like
Yfh1: Yeast Frataxin Homologue 1
ISCU: Iron–Sulfur Cluster Assembly Unifase
SOD: superoxide dismutase
GPX: Glutathione Peroxidase
GPX4: Glutathione Peroxidase 4
GPX1: Glutathione Peroxidase 1
MnSOD: Manganese Superoxide Dismutase
GSH: glutathione
•OH: hydroxyl radicals
NOX: NADPH oxidase
H2O2: Hydrogen Peroxide
ChIP: Chromatin immunoprecipitation
CREB: cAMP Response Element-Binding protein
SP1: Specificity Protein 1
YY1: Yin Yang 1
FoxA1: Forkhead box A1
Gata2: GATA-binding protein 2
C/EBP: CCAAT/Enhancer-Binding Protein
HIF-1α: hypoxia-inducible factor-1α
HREs: hypoxia response elements
HIF-2α: hypoxia-inducible factor-2α
NCOA4: Nuclear Receptor Coactivator 4
CM: cardiomyocytes
CF: cardiac fibroblasts
CpG: Cytosine-phosphate-Guanine
CIRBP: cold-induced RNA binding protein
3′UTR: 3′ Untranslated Region
O2: Oxygen
MVFC: mixed-valent ferroxidase center
TfR: transferrin receptor
TfR2: transferrin receptor 2
Fpn1: ferroportin-1
DMT1: Divalent Metal Transporter 1
IRE/IRP: iron response element/iron response protein
Nrf2: Nuclear factor erythroid 2-related factor 2
NF-κB: Nuclear Factor-κB
AP1: Activator Protein 1
DFO: Deferoxamine
Tf-Fe: transferrin-bound iron
FRDA: Friedreich ataxia
PCD: programmed cell death
ATP: Adenosine Triphosphate
Aβ: Amyloid-β
MDA: malondialdehyde
Cyt *c*: Cytochrome *c*
MAPK: Mitogen-Activated Protein Kinase
I/R: ischemia/reperfusion
ER: endoplasmic reticulum
VDAC: Voltage-Dependent Anion Channel
3-MA: 3-Methyladenine
MPTP: 1-Methyl-4-phenyl-1,2,3,6-tetrahydropyridine
T2DOP: type 2 diabetes-induced osteoporosis
CCCP: Carbonyl Cyanide *m*-Chlorophenyl Hydrazone
DOX: Doxorubicin
LC3: Microtubule-associated protein 1 Light Chain 3
LC3B: Microtubule-associated protein 1 Light Chain 3 Beta
6-OHDA: 6-hydroxydopamine
GMH: Germinal Matrix Hemorrhage
DFP: Deferiprone
BBB: Blood-Brain Barrier
AEE: acute exhaustive exercise
SR-XRF: synchrotron radiation X-ray fluorescence
tBHP: *tert*-butyl hydroperoxide
Mfrn2: Mitoferrin-2
Mfrn1: Mitoferrin-1
KA: kainic acid
AAV: adeno-associated virus
FAC: ferric ammonium citrate
Rb: Retinoblastoma protein
Cdk2: Cyclin-dependent kinase 2
G1/S: Gap 1/Synthesis
AMD: age-related macular degeneration
NRVMs: neonatal rat ventricular myocytes
OPA1: Optic Atrophy 1
mtLIP: mitochondrial Labile Iron Pool
ABCB7: ATP-Binding Cassette Subfamily B Member 7
DISC: death-inducing signaling complex
APAF1: Apoptotic Protease-Activating Factor 1
PIP1: Receptor-Interacting Protein 1
RIP3: Receptor-Interacting Protein 3
TNF-α: Tumor Necrosis Factor Alpha
MLKL: Mixed Lineage Kinase Domain-Like Protein
